# Comparative evaluation of different versions of exposure-free mosquito electrocuting traps and barrier screen trap for monitoring outdoor densities and biting time phenotypes by malaria and filariasis vectors in Tanzania

**DOI:** 10.1186/s13071-022-05549-4

**Published:** 2022-11-11

**Authors:** Victoria Githu, Maneno E. Baravuga, Asiya Mbarawa, Hajirani M. Msuya, Yeromin P. Mlacha, Prosper P. Chaki, Samson Kiware, Nosrat Mirzai, Heather M. Ferguson, Nicodem J. Govella

**Affiliations:** 1 Environmental Health, and Ecological Sciences Department, Ifakara Health Institute, Kiko Avenue, Mikocheni, 78373, Dar es Salaam, United Republic of Tanzania; 2 Faculty of Natural, Mathematical and Engineering Sciences, King’s College London, Strand, London, WC2R 2LS UK; 3 The Pan-African Mosquito Control Association (PAMCA), KEMRI Headquarters, Mbagathi Road, Nairobi, 54840-00200 Kenya; 4 Bioelectronics Unit, University of Glasgow, Graham Kerr Building, Glasgow, G12 8QQ UK; 5 Institute of Biodiversity, Animal Heath and Comparative Medicine, University of Glasgow, Glasgow, UK; 6 The School of Life Science and Bio-Engineering (LISBE), The Nelson Mandela African Institution of Science and Technology (NM-AIST), 447, Arusha, Tengeru United Republic of Tanzania

**Keywords:** Malaria vectors, Trapping methods, Biting times, Mosquito sampling, *Anopheles arabiensis*, *Culex* spp., Mosquito electrocuting traps, Barrier screen trap, Southern-eastern Tanzania

## Abstract

**Background:**

Estimating human exposure to mosquito vectors is crucial for the prediction of malaria transmission and intervention impact. The human landing catch method is frequently used to directly measure estimate exposure rates; however, there has been an increasing shift from this method to exposure-free alternatives, such as the mosquito electrocuting traps (MET) and other approaches. While these latter methods can provide robust and representative values of human exposure and mosquito density, they often still require a human volunteer, which poses logistical challenges. Additionally, in the case of the MET, the early MET prototype (METe) required human volunteers to wear protective clothing that could be uncomfortable. We investigated two alternative trapping approaches to address these challenges by comparing the performance of the METe prototype to: (i) a modified caged MET prototype that offers full protection to users (METc) and (ii) a barrier screen trap (BST) designed to passively sample (host-seeking and blood-fed) mosquitoes outdoors without requiring a human participant.

**Methods:**

The relative performance of the METe, METc and BST were evaluated in a 3 × 3 Latin square field experiment design conducted in south-eastern Tanzania over 12 nights of sampling. The outcomes of interest were the nightly catch of mosquitoes and biting time estimates.

**Results:**

The METc and BST caught similar numbers of *An. arabiensis* as the METe (relative ratio [RR] = 0.76, 95% confidence interval [CI]: 0.42–1.39, *P* = 0.38 and RR = 1.13, 95% CI: 0.63–2.04, *P* = 0.69, respectively). Similarly, the METc and BST caught similar numbers of *Culex* spp. as the METe (RR = 0.87, 95% CI: 0.62–1.22, *P* = 0.42 and RR = 0.80, 95% CI: 0.57–1.12, *P* = 0.199, respectively). All three trapping methods indicated a similar pattern of biting activity by *An. arabiensis* and *Culex* spp., characterized by biting starting in the early evening (18:00–22:00), peaking when people are typically sleeping (22:00–05:00) and dropping off drastically toward the morning (05:00–07:00).

**Conclusions:**

The modifications made to the METe design to improve user comfort and remove the need for protective clothing did not result in an underestimation of mosquito vector abundance nor misrepresentation of their biting time pattern. We recommend the METc for use over the METe design. Similarly, the BST demonstrated potential for monitoring malaria and filariasis vector densities in Tanzania.

**Graphical Abstract:**

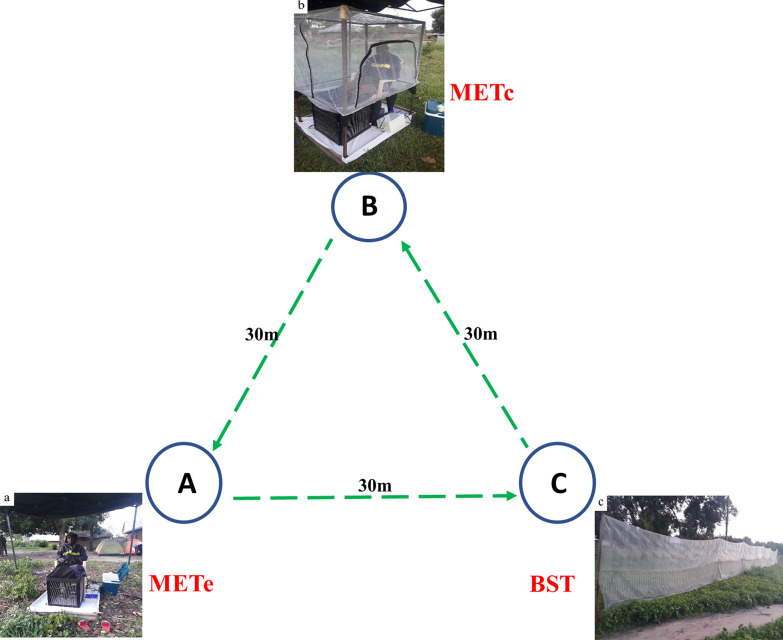

**Supplementary Information:**

The online version contains supplementary material available at 10.1186/s13071-022-05549-4.

## Background

In the efforts toward malaria elimination, effective mosquito sampling methods are critical for monitoring changes in human exposure to mosquito vectors over time and space. Effective sampling is especially useful to highlight gaps in protection with existing front-line vector control tools and to provide insight into how supplementary interventions could be tailored to maximize impact [1, 2]. Unfortunately, only a few methods exist that can reliably and systematically measure the dynamics of human exposure to mosquito vectors (malaria and filariasis) in indoor and outdoor settings, and over different times of the night [3]. The existing gold standard method for the estimation of human exposure is the human landing catch (HLC) [4]. Although the HLC method provides the most direct and epidemiologically relevant estimate of typical human exposure, it has several drawbacks, with the largest one being the ethical concerns it raises due to requiring participants to expose themselves to the bites of potentially infectious mosquitoes that can transmit other infectious diseases in addition to filariasis and malaria, such as dengue, chikungunya, zika and other viral disease for which no prophylaxis or vaccine is currently available [5].

 Recently, a novel tool called the mosquito electrocuting trap (MET) was developed to provide an exposure-free but directly comparable alternative to the HLC [6]. The MET was designed to ensure the protection of participants from mosquito bites during collection by intercepting and electrocuting mosquitoes just before they land on the exposed legs of collectors (in contrast to the HLC where mosquitoes are collected after landing). Similar to the HLC, the MET can be used to quantify human exposure to mosquito bites occurring either indoors or outdoors, which is essential to identify the limits of existing indoor-targeted interventions [7]. In early work, relative to the HLC, the MET caught more *Anopheles gambiae* sensu lato (*An. gambiae* s.l.) from both indoor and outdoor settings in urban Tanzania [8] and similar numbers of *Anopheles arabiensis* outdoors in rural Tanzania [8, 9]. The MET method also reproduced equally relevant estimates of intervention-targetable feeding behaviours of both malaria and filariasis mosquitoes (proportion feeding indoors, proportion feeding at times when most local residents are likely to be sleeping and human exposure occurring indoors) as the HLC [8, 10]. After the initial evaluation in Tanzania, further evaluation of the MET for malaria vector sampling was carried out in Burkina Faso [10]. Here the decision was taken to improve user comfort and provide more standardization and protection by moving from using protective clothing to incorporating a fully enclosed net that covers the entire body. In comparison to some of the studies in Tanzania [8, 9], this modified MET with a protective cage (METc) used in the Burkina Faso study caught proportionately fewer malaria vectors than the HLC; however, it did provide representative estimates of mosquito biting behaviours [10]. It remains unknown whether the somewhat reduced performance of the MET in the Burkino Faso study was due to the introduction of this modification or to other aspects of local vector ecology. Given that all future versions of this MET will likely incorporate this enhanced safety feature of full screened protection, it would be useful to understand the impact of this modification of the trap on performance.

 While incorporating a protective net that fully encompasses users minimizes safety concerns of using METs, both MET methods and the HLC still require human subjects. This requirement makes these methods logistically challenging, complex and difficult to implement at scale. At a routine programmatic scale where only a few essential entomological indicators (e.g. general vector population density, species composition, spatial distribution and seasonality) are useful in decision-making [11], alternative passive methods without the need for human participants would be of great value. Barrier screen traps (BSTs) could be a good choice for this purpose. This passive trap is made out of simple low-cost netting and does not require a human subject to act as bait. Unlike the MET, the BST does not directly measure per capita human biting rates but it can provide a proxy of mosquito density outdoors. BST were initially evaluated relative to HLC in southeast Asia [12], and then in Madagascar [13]. As the performance of mosquito trapping methods can vary between distinct ecological settings [14, 15], local evaluation of the BST method is needed before its implementation for vector surveillance in Tanzania.

 The aim of this study was to field evaluate the performance of the METc and BST relative to the early MET prototype (METe) in terms of sampling sensitivity (number of vectors caught per night), representation of host-seeking activity and species composition of potential mosquito vectors (of malaria and filariasis) in south-eastern Tanzania. In comparing the two MET designs, our aim was to confirm whether the enhanced protection modification of the METc impacted its estimates of human exposure. In contrast, we did not aim to evaluate the BST as an alternative to the METe (as the former is not designed to estimate per capita human exposure), but to assess whether it provides comparable estimates of vector density.

## Methods

### Study location

The field evaluation was carried out at Mgomba Kati village (− 7.951628 S, 38.970745 E), which is located within the Rufiji River basin, south-eastern Tanzania. The area experiences short rains (October– December) and long rains (February–May), with annual rainfall ranging from 800 to 1000 mm [16]. The malaria burden remains high despite the widespread use of insecticide-treated nets (ITNs) [17]. Although this area is among those with the highest malaria burden in the country [18], relatively few entomological studies have been conducted in this area. Previous work indicates that the *An. gambiae* sensu stricto (*An. gambiae* s.s.), *An. arabiensis* and *Anopheles merus* are the major vectors of transmission in this community [19].

### Trap design 

**Fig. 1:**
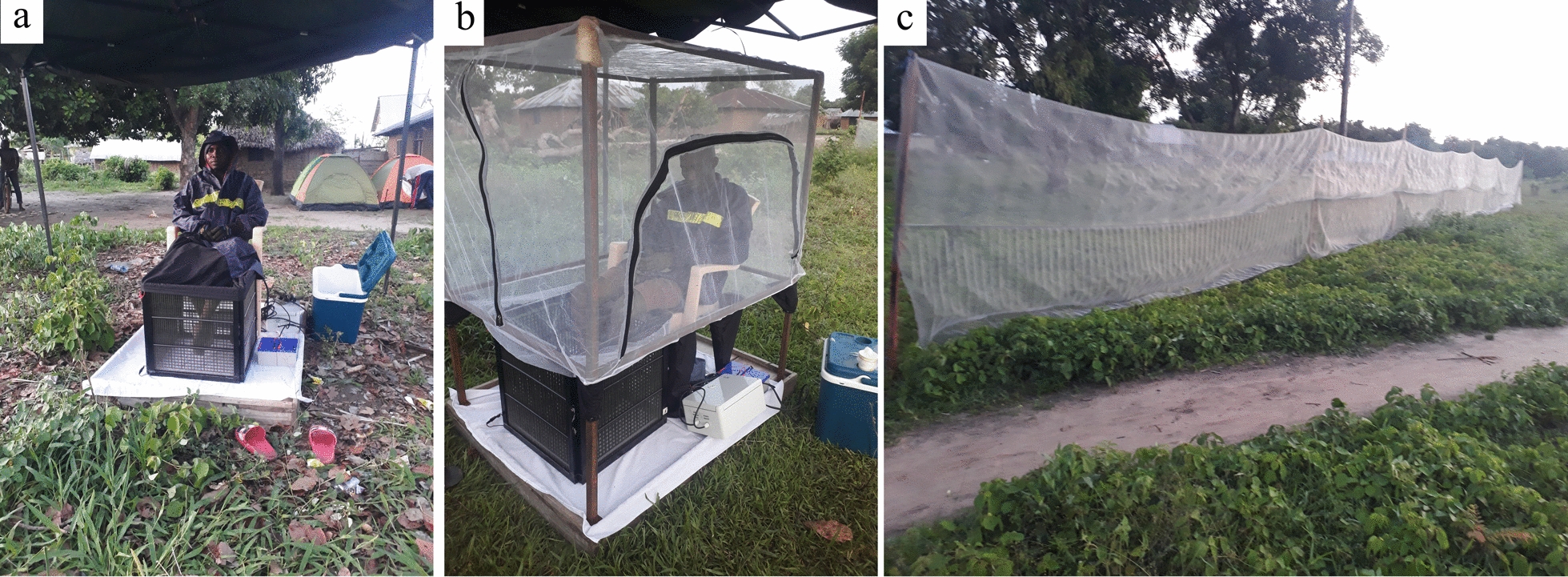
Photographs of the three traps used in the present study. **a** The early mosquito electrocuting trap (METe), **b** the modified mosquito electrocuting trap (METc), **c** the barrier screen trap (BST)

 The METe [5, 6] comprises four 30 × 30-cm polyvinyl chloride panels that are joined to make a square box that encompasses the lower legs of a seated volunteer (Fig. 1a). The panel frames are electrified by means of wires that are spaced 5 mm apart and vertically arranged with alternating positive and negative current. The arrangement of wires is such that mosquitoes attracted to the volunteer are intercepted and killed before they are able to bite the seated volunteer. The MET is powered by two 12-V batteries arranged in series (for details, see [8, 9]). The lower part of the volunteer is fully protected from mosquito bites by the trap, and the upper body part is protected by protective jackets, hats and gloves.

The METc, due to the addition of netted protective cage, was developed by incorporating a new design element to enhance the safety and comfort of the volunteer (Fig. 1b) during mosquito collection. A collapsible cage frame made of four aluminium poles and covered by insecticide-free netting was improvised to fully protect the upper body parts of volunteers [20]. With this design, volunteers are fully protected from mosquito bites. All other aspects of the setup and operation of the METc remained the same as for the METe [8, 9].

 The BST (Fig. 1c) targets both host-seeking and blood-fed mosquitoes. It works by passively intercepting mosquitoes (without the use of adhesive) on their way to either host-seeking, resting or oviposition sites [12]. We used a BST measuring 30 m long and 2 m in height made of whitish polyethylene wire mesh materials. During the end of 15 min of each collection hour, each side of the BST was searched with the aid of a torch by a research team member and any mosquitoes found on the net were collected with a backpack aspirator.

### Study setup design

 Three outdoor catching stations spaced about 30 m apart in an equilateral triangle formation were established within an open field. This field was surrounded by isolated local houses (approx. 50 m apart) along the edge. The METe, METc and BST were positioned in one of the three catching stations on each night that experiments were performed and rotated four times in a serial order through each catching station in a 3 × 3 Latin square experiment design (Additional file 1: Fig. S1). The experiment was conducted over 12 nights of sampling from 17 to 28 November 2020. While traps were rotated through stations, volunteers once assigned to a particular station remained fixed throughout the experiment to minimize any variation associated with volunteers and their catching stations [9, 21]. Mosquitoes were collected hourly (from 18:00 to 07:00) from each trap on each experimental night with catches from each hour placed in hourly labelled paper cups. Sampling was conducted for 45 min of each hour, leaving 15 min for collecting electrocuted mosquitoes on the floor or panels from the METe and METc.

 The catches for each hour from the three mosquito trapping tools were killed, sorted, counted and morphologically identified with the aid of a field microscope as either *An. gambiae* s.l. or *Anopheles coustani* using the keys of Gillies and Coetzee [22] and as *Culex* spp. or *Aedes* spp. All specimens that were morphologically identified as *An. gambiae* s.l*.* were individually stored in 1.5-ml tubes containing desiccated silica gel and cotton wool and subsequently submitted to the laboratory for sibling species confirmation by the PCR assay [23]. Sporozoites were identified by an enzyme-linked immunosorbent assay [24].

### Analyses

 Detailed analysis was restricted to *An. gambiae* s.l. and *Culex* spp. because other mosquito taxa were collected in insufficient numbers and their mosquito count data were over-dispersed. Consequently, generalized linear mixed effect models (GLMM) fit with negative binomial distribution were used to model variation in mosquito abundance (Lme4 package using R statistical software version 3.1.2; R Foundation for Statistical Computing, Vienna, Austria). Each mosquito taxonomic group was analysed separately. The response variable was the nightly catch of *An. gambiae* s.l. or *Culex* spp., with trap type as an independent fixed effect and experimental night as a random effect.

 For comparison of biting pattern estimates, hourly catches in each night were first aggregated into three biting periods: early (18:00–22:00), mid (22:00–05:00) and late periods (05:00–07:00) because of a limited number of mosquitoes in each hourly collection. This categorization of biting time is epidemiologically meaningful, as it relates to the mean abundance of vectors during periods when most people are likely to be unprotected outdoors in the evening before going to sleep (18:00–22:00), when most people are likely to be awake in the morning and unprotected (05:00–07:00) or indoors and protected (22:00–05:00 h) through use of ITNs [25]. Mosquito catches during each biting time category were the response variable, with an interaction between biting time category (early, mid and late) and trap type fit as a fixed effect, and the night of the experiment was treated as a random effect. The predicted mean catch for each biting time category was then plotted in line graphs fitted with standard error (SE) bars for effect size to be visualized. Both models with mosquito abundance as outcomes were fit to a negative binomial distribution to account for overdispersion.

 A simple Chi-square test was employed to explore differences in the composition of mosquito vector groups (malaria and filariasis) between trapping methods. Here, the response variable was the proportion of malaria vectors in the vector sample, as calculated by the number of *An. gambiae* s.l. divided by the combined catch of *An. gambiae* s.l. and *Culex* spp.

## Results

A total of 3410 mosquitoes were collected during the experiment, of which 24.3% (*n* = 828) were *An. gambiae* s.l., 75.3% (*n* = 2570) were *Culex* spp., 0.3% (*n* = 10) were *An. coustani* and 0.1% (*n* = 2) were *Aedes* spp. Of the 828 *An. gambiae* s.l. specimens, 825 (98%) were successfully amplified by PCR, with all of these being confirmed as *An. arabiensis*. Given this result, we use *An. arabiensis* in place of *An. gambiae* s.l. from this point onward. No single individual of the 825 PCR-verified *An. arabiensis* was detected to be sporozoite positive. Slightly more *An. arabiensis* and *Culex* spp. were collected in the METe than in the METc (Table 1), but the difference was not statistically significant for either *An. arabiensis* or *Culex* spp. (Table 2). MET-based traps captured a similar number of *An. arabiensis* and *Culex* spp. as the BST (Tables 1, 2). It should be noted that the majority of *An. arabiensis* collected were unfed, with four, five and 23 blood-fed mosquitoes collected from the METe, METc and BST, respectively.

**Table 1:** Number of *Anopheles arabiensis* and *Culex* spp. mosquitoes captured by the different traps relative to the earlier version of the mosquito electrocuting trap

Trap type	Trap nights (*n*)	Total catch (*n*)	Mean catch^a^	Relative sensitivity^b^
*An. arabiensis*				
METe	12	285	23.75	NA
METc	12	218	18.17	0.76
BST	12	322	26.83	1.13
*Culex spp.*				
METe	12	979	81.58	NA
METc	12	804	67	0.82
BST	12	755	62.92	0.77

*METe* Earlier version of mosquito electrocuting trap (MET), *METc* modified or caged MET,* BST* barrier screen trap,* NA* not applicable

^a^Number of total catch divided by trap nights

^b^Relative to mean catch using METe

**Table 2:** Comparison of the estimated mean catch for each trap analysed using the negative binomial generalized linear mixed model

Trap type	RR [95% CI]	*P*-value
*An. arabiensis*		
METe	1^a^	NA
METc	0.76 [0.42–1.39]	0.38
BST	1.13 [0.63–2.04]	0.69
*Culex spp*.		
METe	1^a^	NA
METc	0.87 [0.62–1.22]	0.42
BST	0.80 [0.57–1.12]	0.19

*CI* Cconfidence interval, *NA* not applicable, *RR* relative rate

^a^Reference group

**Fig. 2:**
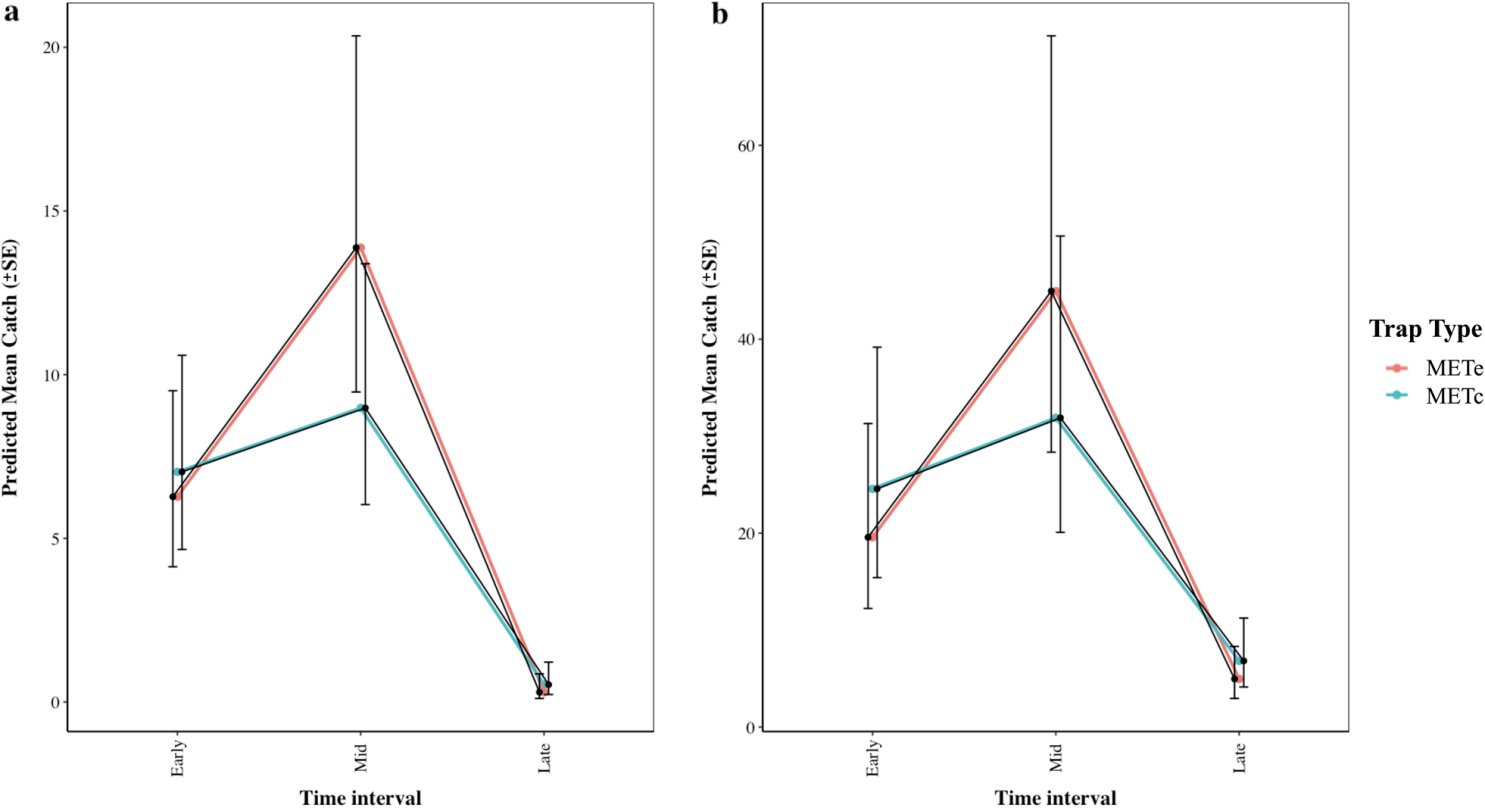
The mean catch biting profile across the sampling time profile for *An. arabiensis* (**a**) and *Culex* spp. (**b**). SE, Standard error

Both MET-based trapping methods yielded a similar pattern of mosquito activity, with biting activity starting in the early period of the evening, peaking during the mid-time period, followed by a steep drop toward the morning (Fig. 2). The mean abundance of *An. arabiensis* and *Culex* spp. in the early biting category was similar in the two MET-based traps (*An. arabiensis*: relative rate [RR] = 1.12, 95% confidence interval [CI]: 0.72–1.75, *P* = 0.61; *Culex*: RR = 1.26, 95% CI: 0.88–1.78, *P* = 0.203). Similarly, the METc and METe generated similar estimates of vector abundance in the late biting category (*An. arabiensis*: RR = 1.58, 95% CI: 0.41–6.1, *P* = 0.51; *Culex*: RR = 1.095, 95% CI: 0.62–1.934, *P* = 0.75). For the mid-time period, estimates of *An. arabiensis* were similar with the METe and METc (RR = 0.577, 95% CI: 0.32–1.052, *P* = 0.072), but the METe caught significantly more *Culex* spp. than the METc (RR = 0.565, 95% CI: 0.35–0.913, *P* = 0.02).

*Anopheles arabiensis* represented 23, 21 and 30% of all mosquitoes caught by METe, METc and BST collections respectively. There was no variation in the distribution of *An. arabiensis* between the MET-based traps (*χ*^2^ = 0.49, *df* = 1, *P* = 0.485). However, the BST sampled a higher proportion of *An. arabiensis* (*χ*^2^ = 25.096, *df* = 2, *P* < 0.0001) than the MET-based traps.

## Discussion

The overall aim of this study was to investigate whether a modification made to the METe enhances user protection without affecting performance in terms of trapping sensitivity and ability to represent biting time. We also sought to assess the potential use of the BST to assess vector density without requiring human subjects [11, 12, 24]. Overall, estimates of the mean nightly biting rate of *An. arabiensis* and *Culex* spp. were similar in the original (METe) and modified prototype (METc), with both traps generating similar patterns of biting time activity. There was only one scenario in which the METc appeared to be less sensitive than the METe: the mean catch of *Culex* spp. host seeking during the mid-period was slightly underestimated in the METc. Despite this difference, the overall performance of the METc was consistent with that of the original prototype, indicating that the modification introduced to enhance and standardize user protection should not reduce sensitivity. Given that this new prototype improves users’ comfort and safety, it is recommended for use over the METe.

 The METc tended to catch slightly fewer *An. arabiensis* and *Culex* spp. than the METe, although the difference was generally not statistically significant. An explanation for this result remains elusive but may be: (i) a sign of a more limited airflow, which would restrict the flow of odour cues around the human subject when protected by a caged net [26]; (ii) due to the cage reducing visual cues from the host; or (iii) due to the net triggering a behavioural avoidance response. However, this variation informs the need to assess the impact of any changes in design or setup to existing trapping methods to ensure similarity and enable comparisons between studies using different versions [27].

 Early work with the METe in urban Tanzania generated estimates of mosquito biting time and human exposure that were consistent with those using HLC [8]. Similarly, consistency between the MET and HLC in terms of biting time estimates was demonstrated in Burkina Faso [10]. The observed inconsistency between MET-based traps in estimates of *Culex* spp. abundance during the mid-period in the present study raises some concern. Notably, we had greater power to detect differences with *Culex* spp. due to their much greater sample sizes, but we may have failed to detect similar differences with *An. arabiensis* due to their smaller sample sizes. Further comparison of these trapping methods over long periods with larger sample sizes and across distinct ecological settings is recommended. However, consistent use of the same version of trap type throughout a surveillance or monitoring period is crucial for time trend comparisons.

 The BST evaluated here provides several unique benefits for mosquito surveillance including: (i) a non-reliance on electricity or a human volunteer; (ii) its simplicity in terms of setup and implementation; (iii) bait-free ; and (iv) low cost. We also demonstrated that it can catch a range of mosquito vector taxa, as well as provide estimates of density that are similar to those obtained from our host-seeking method (MET). This result indicates the BST is not only reliable for mosquito surveillance in rural Tanzanian settings but that it also has the potential for use in large-scale programmes, especially in those studies whose primary emphasis is on monitoring mosquito abundance, spatial distribution and/or species composition rather than on the estimation of human exposure [28].

 The present study has a number of limitations. First, it was relatively short and small scale: it was conducted for 12 nights only, in one village, during the rainy season and outdoors only. Thus, variability in the performance of trapping methods across time and space could not be captured in the analysis. The study site also had a relatively low diversity of malaria vector species (just *An. arabiensis*), thus limiting opportunities to assess how these traps perform for other major malaria vector species, such as *An. funestus,* which dominates malaria transmission in other parts of Tanzania [29]. Also, we did not identify *Culex* to species level, thus limiting information on the relative abundances of species responsible for filariasis transmission. Despite these limitations, the results provide useful insight into the potential use of METc and BST for mosquito bionomics and behavioural surveillance in Tanzania.

## Conclusions

The modification made to the MET did not strongly affect its performance. This new prototype is recommended for use over the original design as it improves comfort and offers complete protection for users against mosquito bites. The BST has shown practical potential for monitoring malaria and filariasis mosquito density in these settings, and may do as well in other settings.

## Supplementary Information

**Additional file 1: Figure S1**. Schematic representation of the experimental design; 3 x 3 Latin square for one complete experimental rotation (round). Blue is the caged Mosquito Electrocuting trap (METc), Orange is the early Mosquito Electrocuting Trap (METe) and the green is the Barrier Screen Trap (BST). **Additional file 1: Fig. S1. **Schematic representation of the experimental design; 3 x 3 Latin square for one complete experimental rotation (round). Blue is the caged Mosquito Electrocuting trap (METc), Orange is the early Mosquito Electrocuting Trap (METe) and the green is the Barrier Screen Trap (BST).

## Data Availability

Access and use of data supporting this article will be made available in Dryad.

## Abbreviations

BST: Barrier screen trap
HLC: Human landing catch
GLMM: Generalized linear mixed model
MET: Mosquito electrocuting trap
METc: Modified or caged mosquito electrocuting trap
METe: Earlier version of mosquito electrocuting trap
RR: Relative ratio
