# Supplementary figures and images for: Comparative evaluation of different versions of exposure-free mosquito electrocuting traps and barrier screen trap for monitoring outdoor densities and biting time phenotypes by malaria and filariasis vectors in Tanzania
Source: Parasit Vectors. 2022 Nov 11;15:420. doi: 10.1186/s13071-022-05549-4 (PMC9652990; doi:10.1186/s13071-022-05549-4)

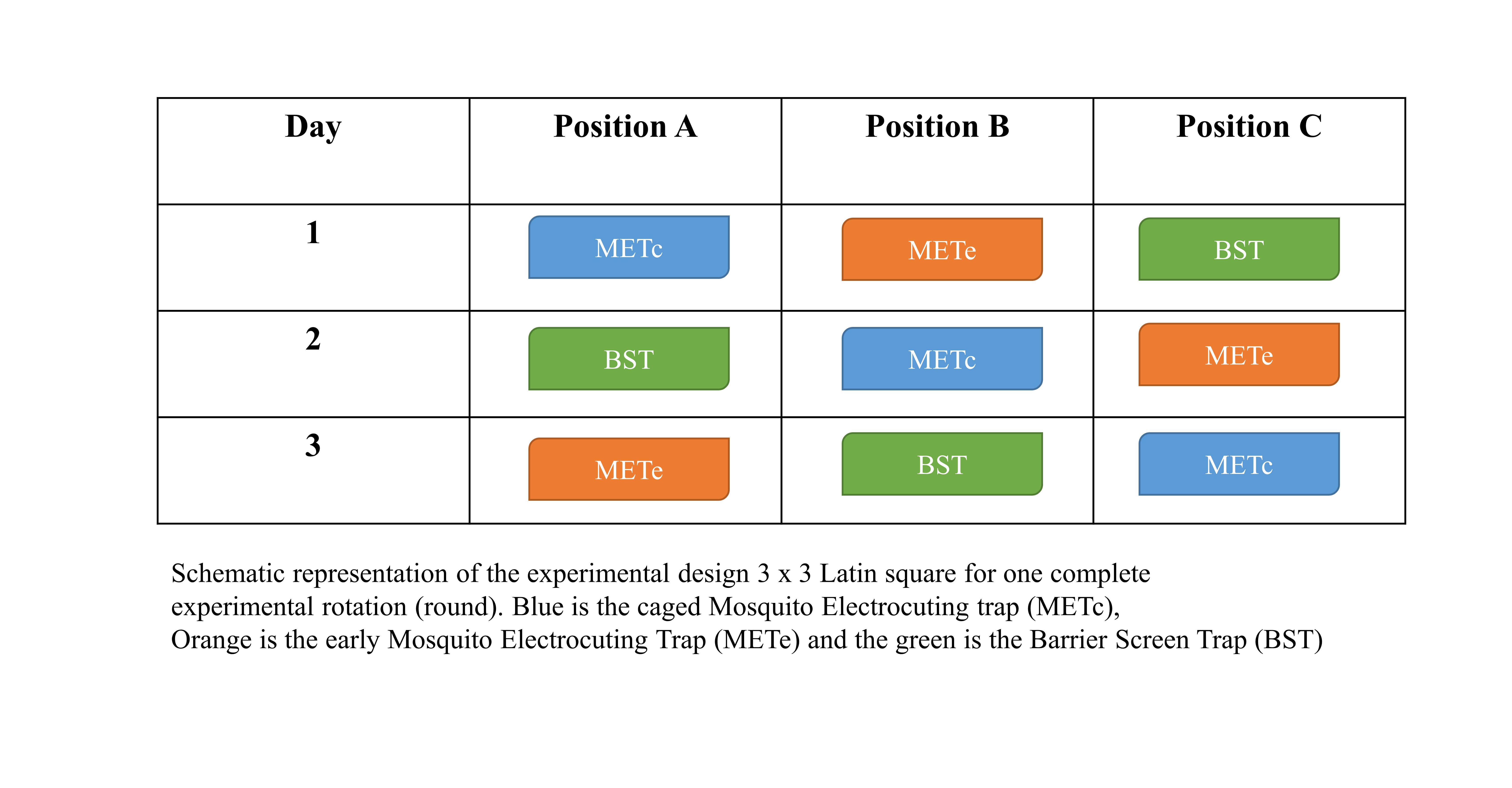

Supplement: Supplementary file 1 — Additional file 1: Figure S1. Schematic representation of the experimental design; 3 x 3 Latin square for one complete experimental rotation (round). Blue is the caged Mosquito Electrocuting trap (METc), Orange is the early Mosquito Electrocuting Trap (METe) and the green is the Barrier Screen Trap (BST). [file 13071_2022_5549_MOESM1_ESM.jpg]
